# Situation awareness errors in anesthesia and critical care in 200 cases of a critical incident reporting system

**DOI:** 10.1186/s12871-016-0172-7

**Published:** 2016-01-16

**Authors:** Christian M. Schulz, Veronika Krautheim, Annika Hackemann, Matthias Kreuzer, Eberhard F. Kochs, Klaus J. Wagner

**Affiliations:** Department of Anesthesiology, Klinikum rechts der Isar, Technische Universität München, Ismaninger Str. 22, 81675 München, Germany

**Keywords:** Situation awareness, Patient safety, Human error analysis, Accident analysis, Anesthesia and perioperative care, Critical care

## Abstract

**Background:**

A loss of adequate Situation Awareness (SA) may play a major role in the genesis of critical incidents in anesthesia and critical care. This observational study aimed to determine the frequency of SA errors in cases of a critical incident reporting system (CIRS).

**Methods:**

Two experts independently reviewed 200 cases from the German Anesthesia CIRS. For inclusion, reports had to be related to anesthesia or critical care for an individual patient and take place in an in-hospital setting. Based on the SA framework, the frequency of SA errors was determined. Representative cases were analyzed qualitatively to illustrate the role of SA for decision-making.

**Results:**

SA errors were identified in 81.5 %. Predominantly, errors occurred on the levels of perception (38.0 %) and comprehension (31.5 %). Errors on the level of projection played a minor role (12.0 %). The qualitative analysis of selected cases illustrates the crucial role of SA for decision-making and performance.

**Conclusions:**

SA errors are very frequent in critical incidents reported in a CIRS. The SA taxonomy was suitable to provide mechanistic insights into the central role of SA for decision-making and thus, patient safety.

## Background

Accurate Situation Awareness (SA) is the indispensable precursor for correct decision-making and action [1–3]. Therefore, it is likely that errors frequently evolve from incorrect SA. Endsley defined SA as “the *perception* of elements of the environment within a volume of time and space, the *comprehension* of their meaning and the *projection* of their status in the near future” [4]. In anesthesia and critical care, the first and lowest level of SA is the *perception* of information that is provided by the patient (e.g., through verbal communication or appearance), monitors, patient charts, communication within the team, anesthesia machine, respirators, and the surgical field (*perception,* SA level I) [1]. On the second level, information is processed in order to comprehend the patient’s state (*comprehension,* SA level II). On the third and highest level, health care providers estimate how the patient will develop in the next minutes and hours (*projection,* SA level III). Generating SA on this level is challenging but important since it allows for proactive management of human and material resources during crisis. To also cover team processes, the framework has been extended defining team SA as “the degree to which every team member possesses the SA required for his or her responsibilities” [5].

Endsley suggested a taxonomy that differentiates between different types of errors on each of the three SA levels (Table 1) [6]. In SA level I (*perception*) errors, relevant information was not perceived (e.g., damaged or missing monitoring equipment as well as communication problems within the health care team can limit the information necessary for adequate SA of an individual). Errors on SA level II (*comprehension*) occur if a complete set of information is not processed correctly resulting in imperfect *comprehension* of the situation. Errors on the SA level III (*projection*) take place if a situation is well understood but the future development is estimated falsely. This error taxonomy has previously been used successfully to systematically analyze incidents in aviation [6, 7].

**Table 1:** Endsley’s taxonomy of Situation Awareness errors

SA level I	Fail to perceive or misperception of information
1.1	Data was not available
1.2	Data was hard to discriminate or detect (e.g., visual barrier)
1.3	Failure to monitor or observe data
1.4	Misperception of data
1.5	Memory loss
SA level II	Improper integration or comprehension of information
2.1	Lack or incomplete mental model
2.2	Use of incorrect mental model
2.3	Over-reliance on default values
SA level III	Incorrect projections of future trends
3.1	Lack or incomplete mental model
3.2	Over-projection of current trends

On each of the levels, errors can occur and SA may be inaccurate, incomplete or even wrong [6]. In SA level I (*perception*) errors information may be unavailable, hard to detect, is perceived incorrectly (although presented correctly), is not observed due to inadequate distribution of attention or simply forgotten. As mental models, automaticity and pattern matching abilities develop over time, individual lack of experience may contribute to a limited capability of adequate and quick information processing resulting in SA level II and III errors

Similar to the Aviation Safety Reporting System, voluntary Critical Incident Reporting Systems (CIRS) have been introduced into anesthesia in several European countries in the last fifteen years [8]. CIRS offer individuals an anonymous platform to report errors and near misses. In a review article, Mahajan emphasized that a systematic analysis of each report is a prerequisite for changes in daily practice [9]. For the purpose of a systematic analysis of errors and error mitigation, error classification [10, 11] and reporting [12, 13] systems have been developed and applied. However, none of them specifically addressed SA errors as a potentially important underpinning mechanism.

Therefore, we determined the frequency of SA errors on the particular SA levels in 200 CIRS cases in anesthesia and critical care. Additionally, a sample of CIRS cases was analyzed qualitatively according to the SA error taxonomy in order to illustrate how SA errors can be associated with wrong decisions, potentially harming the patient.

## Methods

### Study design

After approval of the Research Ethics Board (Technische Universität München, 5770/13, 26^th^ of April, 2013), 200 cases from the German Anesthesia CIRS were analyzed by two independent experts (VK and CS). According to the nature of CIRS, informed consent could not be obtained as the involved individuals remain anonymous. To minimize selection bias, the cases (dating from April to November 2013) were selected in strict consecutive order. For inclusion, a case had to deal with an individual patient and had to be in-hospital and related to anesthesia or critical care. Some reports described general, e.g., structural problems or commented on compromised safety of health care staff. Although the problems described in those cases may significantly impact SA on the level of both individuals and teams, they were excluded as the cases were beyond one individual’s SA and actions. If a case met the inclusion criteria, the experts determined the SA level on which the error occurred as described below.

### Data acquisition

The CIRS used for this study is operated by the German Society of Anesthesiology and Intensive Care Medicine (DGAI), the Agency for Quality in Medicine (ÄZQ) and the Alliance of German Anesthesiologists (BDA). Any health care provider can access the platform without restrictions for both reporting and looking through published reports. The reporters are invited to describe critical incidents and near misses. Prior to publication, trained personnel carefully checks every report for anonymity and content in order to avoid blaming language as well as any possibility to trace back to the persons involved in the case. In this step, also any information and judgment that appears unrelated or unimportant is deleted.

Users report the content of the case in a narrative style of varying length. A part from that, the users can provide data about location (anesthesia, post-anesthesia care unit, intensive care unit, in-hospital transports, code blue team, acute pain management, premedication, other), time point (working day vs. weekend), estimated frequency of this kind of incident (almost daily, weekly, monthly, several times a year, seldom, only this time), professional status of the reporting health care provider (physician, nurse, paramedic, other), his or her work experience (more or less than 5 years), routine vs. emergency case, ASA-classification and whether any medical device was involved. This was recorded to investigate whether the presence and level of SA errors depended on the above-mentioned categorical variables.

### Analysis of CIRS cases for identification of SA errors

In the first step, the critical action of an individual in a case (e.g., the administration of wrong drug, or the absence of an action that would have resolved the problem) was identified. Then, based on the framework of the anesthetist’s SA and Endsley’s definition of SA errors [1, 6], the experts analyzed whether inadequate SA was associated with the decision leading to that action (or non-action, respectively). This required a yes/no response by the experts. If a SA error occurred, also the level (*perception* vs. *comprehension* vs. *projection*) on which the error occurred was determined. If errors occurred on various levels and were related to each other, the experts assigned the error to the most basic level, e.g., pulse oximetry was not used and a health care provider did not comprehend that the patient was desaturating. In this scenario, the causing error was on the level of *perception* and therefore, only this error was coded. If various SA errors occurred independently from each other, the experts only coded the error, which was directly associated with the critical action. The experts analyzed each case independently. If the experts disagreed with respect to the occurrence of a SA error or the level on which the error occurred, the respective case was turned back to the experts for independent re-evaluation. If there remained difficulties to assign the cases to a SA level, consensus was obtained after mutual discussion between three of the authors (CS, VK, KW).

With the aim of illustrating each type of error according to the taxonomy described above, we identified cases that were detailed enough for an analysis of types of errors of the specific SA levels. Each case was translated into English, followed by a brief qualitative analysis in terms of the SA framework and an assignment of the underlying types of error. Additionally, three cases are described where SA was lost and re-gained or where active efforts to gain SA prevented a patient from damage in a rapidly changing situation.

### Statistical analysis

The intended number of 200 included cases permitted to estimate the frequency of a certain case with an accuracy of at least 7 %, which is the confidence interval for the estimated relative frequency. Furthermore, even rare events with a frequency of 1.5 % will be detected at a likelihood of 95 %. Frequencies of SA errors, the respective SA levels and the results of error classification are given as percentage. For an exploratory analysis of correlations between SA errors and categorical data, cross-tables were used with either Chi-square test or Fisher’s exact test when tables contained values of 5 or less. Reliability was calculated using Cohen’s kappa based on the initial assessment of the experts. *P*-values < 0.05 were considered statistically significant. All statistical analysis was done with IBM SPSS Statistics Version 22.0 (IBM Corp., Armonk, New York, USA).

## Results

### Frequency of SA errors

**Fig. 1:**
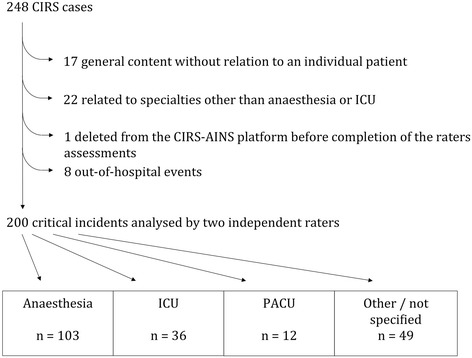
Flow chart. Of 248 cases reviewed, 80.6 % met inclusion criteria. The majority was attributable to anaesthesia (51.5 %), whereas cases on ICU (18.0 %) and PACU (6.0 %) were less frequent. The remaining 49 cases (24.5 %) occurred in locations such as during transports, in code blue teams, during premedication visit or acute pain management. (CIRS = Critical incident reporting system, ICU = intensive care unit, PACU = post-anaesthesia care unit)

248 cases had to be tested for inclusion criteria in order to extract 200 cases that were reviewed by the experts (Fig. 1). 77.5 % of cases were routine cases whereas 21 % were considered to be emergency situations (1.5 % non defined). 24.5 % of cases were reported as singular events, 38.0 % as seldom, 19 % occurred several times a year. The remaining cases (18.5 %) were reported to occur at least monthly. 82.5 % of cases were reported by physicians with 27.9 % of them having less than 5 years of experience. 14.0 % of cases were reported by nurses (with only 10.7 % of them having less than 5 years of experience). Median of ASA score was 2.

**Fig. 2:**
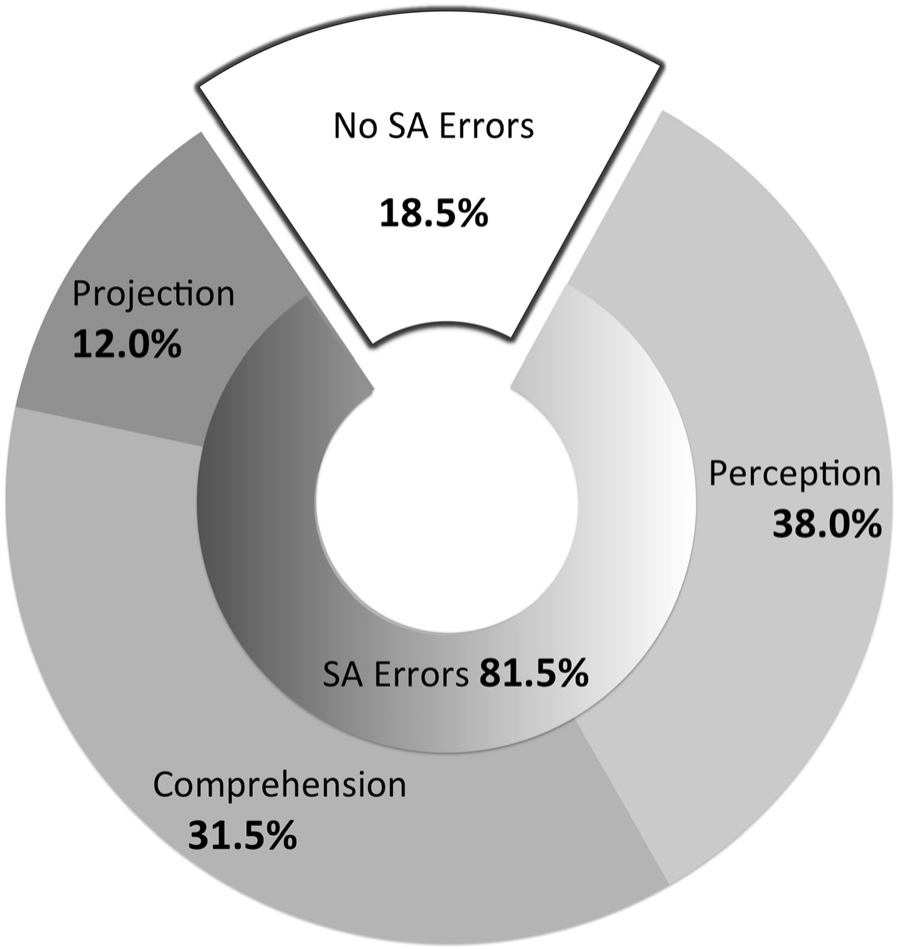
Distribution of SA errors on the levels perception, comprehension and projection

SA errors were identified in 163 cases (81.5 %). The large majority was attributed to the levels of *perception* and *comprehension* (Fig. 2). Both experts agreed in 90 % of cases whether an SA error was present or not (Cohen’s kappa 0.69). With respect to the level on which the error occurred, agreement was present in 66.9 % (Cohen’s kappa 0.48) of cases.

Cross tables revealed that the frequency of SA errors and the level on which they occurred were independent from the location (anesthesia, ICU, PACU, other/not specified) and from other categorical data (time point, estimated frequency, professional status, work experience, routine vs. emergency case, ASA-classification and whether a medical device was involved into the incident [data not shown]).

### Qualitative analysis of SA errors

In the following, cases with SA errors are described (see Table 2 for details). Case 1 refers to information that was either missing due to insufficient communication or that was simply forgotten. Cases 2–4 are examples of barriers that physically preclude from information to become (acoustic and visual) sensory input (e.g., “After putting the drapes, the access to both peripheral iv lines was hampered”). In case 3, specifically, “small fonts” on a display prevented from a more timely recognition of the fact that a propofol syringe pump ran with remifentanil and vice versa. In case 5, relevant information was missed due to the decision not to use a patient monitor. Case 6 describes the mis*perception* of drug labels resulting in the decision to use hydroxyethyl starch to keep open an arterial line. The reporting individual identified a look-alike problem of drugs (“both look similar”) which is a problem addressed extensively elsewhere [14]. In these cases (1–6), the involved individuals did not perceive relevant information and consequently, they did not comprehend important aspects of the situation and, as a result, wrong or no decisions were made.

**Table 2:** Fifteen examples of SA errors

Case number	Case description	Analysis from the SA perspective	SA level
1	An anesthesiologist took over a patient who had undergone massive transfusion including catecholamine therapy. He reports to have received a *“detailed handover”* and that his job was to finish the procedure and to transport the patient to ICU. Just before leaving the OR he replaced an empty infusion bag with a new one in order to continue volume replacement. Immediately afterwards, the patient’s suffered from ventricular arrhythmia and the systolic blood pressure increased to 250 mmHg. *“During check of the i.v.-lines I noticed that the adrenaline syringe pump had been connected to the central line by two extension lines type Heidelberger. Obviously they had filled with highly concentrated adrenaline which was administered unintentionally during volume resuscitation.”*	The anesthesiologist was not aware about a significant amount of adrenaline in the lines. Possibly, the hand-over, which he felt to be “detailed”, did not include information about this fact (SA-I). Alternatively, he may have forgotten this information in face of a complex situation where gaining complete SA in short time is challenging for someone who had not been involved until this moment.	SA I
			data not available *or* memory loss
2	*“The code blue physician does not hear the beeper. The beeper turns off after a certain time. The causes are a significant noise exposure on the ICU and the high frequency of phone calls.”*	The code blue physician did not perceive the alarm (SA-I). The reporting individual mentions acoustic barriers on one hand and high workload on the other hand as causes.	SA I
			hard to detect
3	*“For economic reasons, sometimes, nurses program the syringe pumps. In this case a syringe pump programmed for propofol ran with remifentanil and, accordingly, it ran too slow. […] The only striking point was that we had propofol in the remifentanil line repeatedly and despite high infusion rates, we still had the first syringe of remifentanil after hours. Having a closer look we were able to recognize propofol in small fonts on the display whereas remifentanil was indicated on the syringe label.”*	It largely remains unclear why the nurse allocated the drugs incorrectly. Assumingly some information (syringe content or pump program) has been forgotten. However, the reporting individual clearly states that important information was displayed in small fonts hindering a fast and quick recognition of the content of syringe pumps (SA-I).	SA I
			data hard to discriminate
4	*“After putting the drapes, the access to both peripheral iv lines was hampered. During team-time-out one of the surgeon leant against the arm compressing the iv lines while the anesthesiologist paged through the patient’s health record […] so that the anesthetics entered the infusion bag of the crystalloids. During skin incision the patient showed increase of heart rate and moved the arms. Then, we switched the administration of anesthetics to the other iv access.”*	Important visual information from iv lines (obstruction) was not perceived due to a visual barrier (drapes). Furthermore, the visual attention was directed to the patient’s health record during team time out. It remains speculative why a non-return valve had not been used and whether the use of such a valve had resulted e.g., in high-pressure alarms in the syringe pumps (SA-I).	SA I
			hard to detect *and* failure to observe
5	After uneventful anaesthesia the patient was transferred to another location. There, the first systolic blood pressure assessed was 60 mmHg. *“In this OR a transport monitor does not exist. The short transfer regularly is done without monitoring. Every time a monitor is required, we have to get it from elsewhere which is time-intensive.”*	The case reveals structural problems as a monitoring device is not easily available and the anesthesiologists avoid time delays in face of assumingly uncomplicated cases. As a result, important information is missed (SA-I).	SA I
			failure to monitor
6	*“To keep open an arterial line, HES [hydroxyethyl starch] was used instead of saline. Both look similar but HES is an emergency substance so that it should be stored in a different place.”*	As both infusions look similar (look-alike problem), the information was correct but obviously misperceived (SA-I).	SA I
			misperception
7	*“A patient is transferred to ICU with several syringe pumps including a pump for TIVA [total intravenous anaesthesia] that had been equipped with a catecholamine. The ICU personnel are not familiar with that type of pumps. […] Unintentionally, the patient got a high bolus.”*	A health care provider works with a syringe pump he is not familiar with. Although all the dynamic information is present (rates, drugs, indication), the individual applies an incorrect mental model of the pump’s operating mode and thus, he lacks of comprehension (SA-II).	SA II
			use of incorrect mental model
8	*“two oral drugs […] had been given via the central venous line instead of the gastric tube.”*	Assumingly, all the relevant information (e.g., package insert, drug orders) was present, but the individual lacks of a mental model with respect to how these drugs are administered (SA-II). As a result he does not comprehend that these drugs have to be administered in another way.	SA II
			use of incorrect mental model
9	“*[…] On the third postoperative day […] the epidural was stopped. On the next morning, the anesthetist cannot visit the patients due to concurrent obligations. During the evening visit, the anesthetist noticed that ropivacaine was re-started but that it was connected to a peripheral venous line. The infusion was stopped immediately.”*	Assumingly, all the relevant basic information was present: drug, patient and indication (SA-I). But the information was not properly integrated, due to missing knowledge or the use of missing or an incorrect mental model (SA-II). If someone is confronted with a set of information he can’t process due to missing contextual contents in the long-term memory, he will probably ask for assistance. If an incorrect model is used, he won’t recognize the error as long as there is no additional information such as visible adverse effects.	SA II
			use of incorrect mental model
10	*"During TIVA a change of the syringe (remifentanil) was pending. The syringe had been prepared by the nurse (50 ml, clear solution). The label “remifentanil” and the ampoule lied besides the syringe. The nurse told to the anesthesiologist that the remifentanil syringe was prepared. The anesthesiologist changed the syringe; in the following minutes, the patient shows tachycardia and high blood pressure, deepening anaesthesia is without success. When the nurse came back, she asked if the anesthesiologist had added the remifentanil to the prepared syringe. As it turned out, the communication […] was unclear and stated a potential danger for the patient.”*	The anesthesiologist incorrectly assumed a syringe to be correctly prepared (SA-II). Visible information (the ampoule next to the syringe) was not perceived or not integrated in order to come to the conclusion that the syringe contained purely saline. Additionally, the reporting individual identified a lack of information resulting from unclear communication as the cause.	SA II
			over-reliance on default values
11	*“A critically ill patient with complex pains, who was visited by pain physicians for 4-fold analgetic medication. During change of syringe pump, ketamine is administered in wrong dosage, 50 mg/ml instead of 1 mg/ml is administered, as it is usual for sedation. During shift change the error is recognized. […] The patient was awake throughout the case […] but suffered from headache.”*	The nurse who changed the syringe prepared the dosage as usual (assuming standard values), despite differing information from the medication order as indicated through the fact that this was recognized during shift change. This may have happened through an over-reliance on default values (SA-II) although additional information was available that would have resulted in a different action (preparing the correct dosage).	SA II
			over-reliance on default values
12	*“During thoracic surgery (VATS lobectomy) the suction catheter was introduced too deep in the tracheal part of the double-lumen tube. […] Lobectomy is performed using a stapler. The suction catheter could not be removed for checking for leakiness […]. As a cause, the stapler had fixed the suction catheter. An anterior thoracotomy was performed […] and the suction catheter was removed successfully.”*	The anesthesiologist, assumingly, was aware about the surgical procedure to be performed (use of stapler). Additionally he had the information about the suction catheter as he himself had inserted it. This information has not been integrated properly as he relied on his experience from prior situations where removing the device was always without problems and long-term memory content such as a mental model or prototypical situations suited to successfully integrate the basic data was not used or not present. As a result, also a problem on the level of projection emerges as an anterior thoracotomy had to be performed unexpectedly.	SA II
			lack of or incomplete mental model
13	*“A surgeon indicated emergency surgery. There is no written information about patient history and it is impossible to get the information orally [from the patient]. The patient is assessed clinically, an old scar from tracheostomy is visible which indicates possible intubation problems. The anesthesiologist put himself under pressure and induces anaesthesia without investigating the background or consulting the admitting hospital. A rapid sequence induction is performed. Intubation with a 8.0 size tube is not possible, bag mask ventilation works, a 7.0 mm is not introducible as well, and a laryngeal mask (4 and 5) is not tight so that adequate ventilation is impossible. Finally, another physician successfully intubates.”*	Unexpectedly, the anesthesiologist ran into intubation difficulties, indicating an error on the SA level of projection (SA-III). This is supported by the retrospective statement that he worked under avoidable time pressure and that, as a consequence, search for additional information was omitted (SA-I). Regardless of the fact whether the simple presence of a scar from tracheostomy should prompt the preparation for difficult airway management, a mental model that integrates the basic data (tracheostomy in the past) to SA on the level of projection “expected difficult intubation” was absent (SA-III).	SA III
			lack of or incomplete mental model
14	*A patient is scheduled for hip replacement. […] Until the use of palacos bone cement everything went fine. […] Immediately after inserting palacos bone cement, end-tidal CO*_*2*_ *drops from 37 to 13 mmHg. Oxygen saturation does not provide values. At the beginning, a sinus tachycardia of 140 bpm is noticed, quickly followed by deformed QRS complexes. Heart rate drops to 20 bpm. Cardiopulmonary resuscitation is initiated immediately. The working hypotheses are air embolism, fat embolism and allergic reaction.*	An unexpected deterioration due to the use of palacos bone cement is described (SA-III). A dramatic change of vital parameters is the basic information (SA-I) that results in a re-evaluation of the situation. As a consequence, the anesthesiologist comprehends that cardiopulmonary resuscitation is required (SA-II). Additionally, based on basic information, possible causes are discussed.	SA III
			over-projection of current trends
15	A geriatric patient with dementia is transported to the emergency department. He has a visible laceration on the head after having fallen out of the bed. The laceration was sutured and a CT scan ordered in face of increasing somnolence. “*A medical student saw that nobody had placed a cervical collar and that the patient complaint about pain when the head was positioned for suturing. He did not communicate his observation […]. The scan showed a facture of atlas and axis.”*	There are relevant cues that indicate the possibility of a lesion of the cervical spine (fall, laceration on head, increasing somnolence, pain during movement of the head). The reporting individual emphasizes that the team did not comprehend the possibility of a spine lesion that is, they either did not possess over the mental model that allowed for meaningful integration of the information mentioned above (SA-II) or they simply did not perceive some piece of information, e.g., pain during movement of the neck (SA-I).	SA I
			failure to observe
			SA II
		Another point refers to a lack of communication as the medical student did not speak up (Team SA). Communication can refer to the SA level of comprehension (e.g., “we cannot rule out a spinal lesion, therefore cervical collar makes sense”) or to the level of perception (e.g., “every time the patients head/spine is moved, the patient complaints about pain”).	missing mental model
			TEAM SA

Fifteen cases during which SA errors led to errors or near misses. SA-I refers to the level of perception, SA-II to the level of comprehension, SA-III to the level of projection, respectively

In cases 7–12 the information was complete but processed incorrectly. In case 7, a health care provider was not familiar with the operating mode of a pump, nevertheless he operated the pump and unintentionally administered a bolus. A similar problem occurred in the cases 8 and 9 where drugs were administered in the wrong way. In SA terms, incorrect mental models were applied. Cases 10 and 11 illustrate incomplete comprehension of a situation due to the over-reliance on default values. In case 10 an anesthesiologist incorrectly assumed that a syringe was ready to use. In case 11 the drugs were prepared as usual in spite of a drug order that differed considerably from routine. In case 12, presumably, the anesthesiologist was aware that a lobectomy was to be performed using stapler technique. Nevertheless, he had introduced the suction catheter into the tracheal lumen of the tube. These two points were not brought together, as this set of information did not fit into preformed long-term memory content indicating a lack of a mental model.

Cases 13 and 14 provide examples of how anesthesiologists unexpectedly are confronted with a problem or a deterioration of the patient, indicating incomplete awareness on the level of *projection*. In case 15, a team member (a student) did not inform the rest of the team about his situation awareness (SA level I and II) with respect to a patient in the emergency department. As a result, not every team member possessed the SA needed for her/his responsibilities (Team SA).

In contrast, the cases 16–18 (Table 3) illustrate how the search for additional information and continuous re-evaluation of the situation is crucial to maintain SA. In case 16, an additional routine check of basic data prevents a transfusion incident whereas in case 17, a situation (esophageal intubation) is not comprehended until additional information (bronchoscopy) is sought and processed. Case 18 is an example of a situation in course of a transport of a patient for computed tomography where the basic information changes very quickly and in an unexpected manner. The persons involved were able to dynamically adapt their SA and this enabled them to make the right decisions in face of at least two life-threatening problems.

**Table 3:** Three Examples of re-established SA

Case number	Case description	Analysis from the SA perspective
16	*“The nurse brings red blood cell [RBC] concentrate into the OR. During check we noticed that the RBC data sheet does not match the patient (same blood group, wrong patient). […] The nurse had to care for too many OR so that she did not check for patient’s name.”*	Although the mismatch had been identified just before transfusion, the reporting individual claims that there was a failure to perceive important data when taking the RBCs out of the fridge (SA-I). According to the report, this was caused by excessive workload. Another check prevented from possibly negative consequences.
17	*“Intubation with a double-lumen tube after visualizing the glottis. During bag ventilation, no end-tidal CO*_*2*_ *was measured. Assuming bronchial obstruction, forceful attempts to inflate the lungs results in small oscillations of the CO*_*2*_ *curve. […] Bronchoscopy by the attending called in reveals esophageal intubation. Meanwhile, SpO*_*2*_ *had dropped. A single-lumen tube was placed for oxygenation and after a few minutes, this was replaced by a double-lumen tube using tracheal tube introducer without problems.”*	After a normal intubation, there is no end-tidal CO_2_. As this combination of basic data is contradicting (and therefore not comprehended, SA-II), a re-evaluation including the search for additional information (bronchoscopy) is prompted (SA-I) with the aim for understanding the situation.
		After getting SA on the comprehension level (SA-II), the anesthesiologist decides to preferably use a single-lumen tube for safe oxygenation in order to avoid on-going intubation difficulties (SA-III).
18	*[…] Due to respiratory distress, the patient was intubated. […] During a transport for CT scan of the thorax, the patient became haemodynamically unstable requiring an increasing dosage of noradrenaline. The initial scan showed pneumothorax corresponding to the clinical assessment. There was the indication for placing a drain quickly. During puncture, the patient developed a haemodynamically highly relevant tension pneumothorax (HR > 180 bpm, blood pressure 90/40 with noradrenaline). Unfortunately, the needles available were not sufficiently long and thick. Therefore air was removed using a drain. Afterwards the blood pressure stabilized but tachycardia remained, later on switching to ventricular tachycardia. On the code-blue trolley there was only an automated external defibrillator so that for cardioversion a defibrillator had to be retrieved from an ICU several floors above. The sinus rhythm, achieved thereby, improved the situation significantly […].*	In face of deteriorating vital parameters, the team realizes that a pneumothorax is the most probable cause following the result of the initial scan. Before the puncture, additional basic information is collected by a clinical assessment (assumingly auscultation) to confirm the diagnosis.
		After successful puncture, the basic data (vital parameters) change favorably but do not reach normal values. After integrating additional basic information on the monitor (the ECG waveform), the diagnosis of a ventricular tachycardia (SA level II) is made and the need for cardioversion is recognized (decision-making). As the AED is not suitable for cardioversion (long-term memory content), the team decides to retrieve a defibrillator from elsewhere.

Three cases where SA was re-established. SA-I refers to the level of perception, SA-II to the level of comprehension, SA-III to the level of projection, respectively

## Discussion

Quantitative reports about the frequency of SA errors in health care have been absent so far. This study provides evidence that SA errors are frequent in those critical incidents cases that were reported voluntarily in a CIRS. This is in accordance with findings from aviation where the frequency of SA problems in operational errors ranged between 59 % and 88 % [15]. In this exploratory approach, the frequency of SA errors and the distribution between the SA levels did not differ significantly between anesthesia, ICU, PACU and others. Furthermore, it was independent from the estimated frequency, professional status and experience of the reporting individual. Only in a minority of cases with critical incident (18.5 %), no critical action was identified or tracing down from a critical action to an associated SA error was not possible. Several other studies in health care qualitatively examined the role of SA in different settings such as during diagnostic errors in primary care [16], in nurses during medication administration [17] and during object transfer in the operating theatre [18], and the interdisciplinary management of patient risk in inpatient settings [19]. However, no study provided quantitative data about the frequency of SA errors in any setting so far.

In contrast to an analysis of 143 incidents reported in the voluntary Aviation Safety Reporting System (76.3 % SA level I errors, 20.3 % SA level II errors and 3.4 % SA level III errors) [7], we found less errors on the level of *perception* and more errors on the levels of *comprehension* and *projection*. As errors on the SA levels II and III necessarily involve long-term memory content for the processing of basic data [1], this may be due to a lack of training and experience. This is not surprising because staffing in health care is, in contrast to commercial aviation, less standardized with respect to quality and quantity. Another domain-specific explanation is that both, pilots and air traffic controllers work in highly standardized settings and they always utilize the same set of information (e.g., pilots always have an altimeter available). An anesthetist, in contrast, has to actively seek for information (e.g., through observing the surgical field or through communication) and must decide whether an additional source of information (e.g., arterial line) is essential for successful management of the case and whether invasive measures are justified.

The cases for the qualitative analysis were selected purposefully with the aim of providing examples for each of the type of error in Endsley’s taxonomy. We intended to demonstrate that errors on the more basic levels obligatorily cause errors on the more advanced levels and finally lead to wrong or missing decisions that in turn compromise patient safety or even cause patient harm. This relationship was reflected by cases where wrong decisions base on a lack of SA and cases in which SA abilities in dynamic situations resulted in adequate management of a problem. In this sample, the taxonomy was suitable for providing mechanistic insights in the analysis of particular cases. In one qualitative study on diagnostic errors in primary care, the SA framework was also found to be useful for a descriptive evaluation [16].

An important pre-condition, however, for breaking down the causality from critical action to decision-making and further to SA with types of errors on the respective SA levels, was that the reports had to be detailed enough. That was seldom the case and is also reflected by the fact that the *a priori* accordance of the raters decreased with increasing depth of analysis. For example, on the level of individuals, it can be difficult to decide whether an error occurred due to the missing arterial line (information not available, *perception*) or whether the error occurred due to the decision not to place an arterial line (incomplete, an incorrect or an absent mental model, *comprehension* or *projection*). On the level of teams, the reports were not detailed enough to detect possibly missing team interactions such as SA cross-checking. Therefore, this approach does not necessarily cover ineffective teamwork where SA is considered to be an important determinant for performance [20]. For that purpose, other databases are required. Finally, our approach only identified one individual SA error per case, specifically the error which led to a wrong decision and consequently to the critical action. As even one single SA error can be multifactorial, this is not in contrast to Reason who used the Swiss cheese model to illustrate that often more than one defense barrier has to fail before an error occurs [10]. In this context it is important to remark that in our study the SA construct was used to analyze SA errors that occur in individuals rather than to analyze SA errors that are caused by individuals. In hindsight it is easy to determine which SA the individual should have possessed as “people holding a false belief do not know it is false” [21]. To prevent the individuals from being blamed, however, it is imperative to assess the information available in the respective situation and the conditions under which the individual worked. Furthermore, the individual’s long-term memory content and the extent to which it is available for data processing, e.g., under conditions of high workload, has to be considered. Long-term memory content is integral for building SA on the levels II and III and cannot be influenced by the individuals in course of a critical incident [1]. Obviously, this concept is helpful for a better understanding of decision-making but, in the far majority of cases, it does not allow for drawing conclusions with regard to the individual’s responsibility to gain and maintain accurate SA [21, 22].

There is a large body of literature about human errors. Reason promoted a paradigm change when he suggested shifting the focus from the individual to the system, in order to continuously improve the conditions under which humans work and thus, to reduce errors and enhance reliability in health care [10]. Another important step was to systematically analyze the frequency and quality of critical incidents, the first among them the Closed Claims Project of the American Society of Anesthesiologists [23] and the Australian Critical Incident Monitoring Study [12]. Both have led to a number of changes in daily practice [24]. Two recent interventions, a new drug administration system [25] and the World Health Organization’s Surgical Safety Checklist [26], resulted in reduced mortality and adverse events. In our view both interventions intend to enhance SA: The drug administration system provides additional auditory input on the level of *perception*, includes cognitive aids (e.g., it remembers to administer antibiotics within 15 min after skin incision) and intends to reduce workload by an electronic health record, which permits observing the patient more intensively [25]. The Surgical Safety Checklist promotes Team SA by forcing communication through a standardized form [1, 26].

Some important limitations are inherent to any analysis based on CIRS. Evidently, the reports are of limited length, reported chronologically, and explanatory elements often limit to the medical and technical context. Accordingly, reporting individuals rarely consider human elements such as non-technical skills. Recently, Haller et al. reported clinical factors that were associated with non-use of the system such as regional anesthesia, emergency procedures and severe complications with a high risk of litigation [27]. Furthermore, there are hindering factors such as the individual’s role in an incident, and a lack of safety culture on the individual as well as on the institutional level. Not all hospitals and departments agree to publish their cases. Additionally, with the aim of ensuring the reporter’s and patient’s anonymity, in some cases the CIRS personnel may be forced to delete also important information including whether or the extent to which a patient may have been harmed. Taken all together, in case-to-case decisions, only a minority of the real critical incidents is published in CIRS and therefore, the results are not a valid picture of the real quality and quantity of critical incidents. Nevertheless, in face of the frequency of SA errors in this subsample and the possibility to mechanistically describe their consequences, the authors suggest that the SA framework is a promising approach to enhance our knowledge about the development of human error in health care. However, more representative databases are required. For the SA framework, it still has to be shown that the approach of analyzing incidents is suitable to 1) systematically and completely detect causal relationships between SA, decision-making and performance, 2) to identify factors that contribute to these SA errors, and 3) to derive promising interventions that successfully address frequent sources of SA errors.

Another technique designed to develop a thorough understanding of domain-specific SA requirements and cognitive decision-making processes is the Goal-Directed Task Analysis [28]. It is a form of cognitive task analysis that focuses on individual’s goals, decisions, and information requirements that support those goals and decisions. Based thereupon, future promising steps include the development of more user-centered monitoring systems that, for example, integrate basic data in order to provide information that directly enhances individuals’ SA on the more advanced levels [29], e.g., by suggesting a diagnosis. In some locations, structural alterations that not only address the availability of but also the use of monitoring systems may be a relatively easy first step. Last but not least, new training approaches should be directed to foster the individual skills needed for developing SA adequately and quickly.

## Conclusion

In conclusion, SA errors are very frequent in critical incidents reported in the German CIRS for anesthesia and critical care. The SA framework is suitable to provide mechanistic insights into the development of critical incidents. However, prior to identifying promising interventions for the reduction of SA errors, further robust analysis tools and representative databases for the health care system are required.

## Abbreviations

ÄZQ: Agency for Quality in Medicine
BDA: Berufsverband Deutscher Anästhesisten (Alliance of German Anesthesiologists)
CIRS: Critical Incident Reporting System
DGAI: Deutsche Gesellschaft für Anästhesiologie und Intensivmedizin (German Society, of Anesthesiology and Intensive Care Medicine)
ICU: Intensive Care Unit
PACU: Post anesthesia care unit
SA: Situation Awareness
